# Enterprise Integration and Interoperability for Big Data-Driven Processes in the Frame of Industry 4.0

**DOI:** 10.3389/fdata.2021.644651

**Published:** 2021-06-03

**Authors:** Alexandros Bousdekis, Gregoris Mentzas

**Affiliations:** Information Management Unit (IMU), School of Electrical and Computer Engineering, National Technical University of Athens (NTUA), Athens, Greece

**Keywords:** conceptual modeling, data analytics, enterprise architecture, data management, smart manufacturing, predictive maintenance

## Abstract

Traditional manufacturing businesses lack the standards, skills, processes, and technologies to meet today's challenges of Industry 4.0 driven by an interconnected world. Enterprise Integration and Interoperability can ensure efficient communication among various services driven by big data. However, the data management challenges affect not only the technical implementation of software solutions but the function of the whole organization. In this paper, we bring together Enterprise Integration and Interoperability, Big Data Processing, and Industry 4.0 in order to identify synergies that have the potential to enable the so-called “Fourth Industrial Revolution.” On this basis, we propose an architectural framework for designing and modeling Industry 4.0 solutions for big data-driven manufacturing operations. We demonstrate the applicability of the proposed framework through its instantiation to predictive maintenance, a manufacturing function that increasingly concerns manufacturers due to the high costs, safety issues, and complexity of its application.

## Introduction

Enterprise integration and interoperability has been established as a scientific challenge of outmost importance (Panetto et al., 2016), especially in the frame of the emergent technologies of the Internet of Things (IoT), big data, and Artificial Intelligence (AI). This trend has inevitably affected the manufacturing domain, which is currently feeling the approach of the fourth industrial revolution and new approaches on data management and interoperability (Romero and Vernadat, 2016; Fraile et al., 2019; Zeid et al., 2019). However, the technical challenges for the transition to Industry 4.0 are strongly related to those of the whole business environment. To this end, the Enterprise Integration and Interoperability research domain should be adapted to smart manufacturing requirements in order to facilitate the design and implementation of Industry 4.0 solutions and enable the stakeholders to meet their expectations.

In this context, the increasing size of big data poses additional challenges and asks for novel techniques of software engineering for their design, analysis, and development. The existing literature has been contributing to these challenges, mainly focusing on the development of architectures for addressing lower level interoperability challenges of Industry 4.0, such as distributed storage, data aggregation, and service orchestration, as well as big data infrastructures. On the other hand, the literature is rich on software frameworks, often overlapping or controversial to each other, something which has resulted in the design of *ad-hoc* and complex architectural big data solutions (Davoudian and Liu, 2020).

Arguably, the most widespread and influential architectural framework in the manufacturing domain is RAMI 4.0. Overall, in the literature, there are only a few case studies that follow the RAMI 4.0 model, and even fewer not requiring much effort to reach the level of practical implementation (Pisching et al., 2018). However, the key issue of any design and system development in the context of Industry 4.0 is the proper implementation of RAMI 4.0 in various manufacturing operations and the definition of appropriate sub-models for individual aspects and processes according to the technical background of Industry 4.0 (Zezulka et al., 2016; Moghaddam et al., 2018; Bousdekis et al., 2019a). To this end, there is the need for architectural frameworks that will enable the systematic design and development of Industry 4.0 solutions so that they tackle the big data-rich, complex, and uncertain manufacturing environment in a holistic way.

In this paper, we propose an architectural framework for the design and development of software solutions for big data-driven processes in Industry 4.0 in order to deal with the integration, interoperability, and data management challenges of the manufacturing environment. The proposed framework is based upon three pillars: Enterprise Integration and Interoperability, big data processing, and Industry 4.0. The synergies among them derive from the requirements that guide the design of the proposed framework. Then, the framework is instantiated to predictive maintenance and serves as the basis for the development of a predictive maintenance platform. The platform was applied to three business cases according to their requirements. In this paper, we describe its deployment and present the evaluation results of a case study from the steel industry.

The rest of the paper is organized as follows. Section Literature Review provides a literature review on the three pillars of the proposed framework, i.e., Enterprise Integration and Interoperability, big data processing, and Industry 4.0. Section The Proposed Architectural Framework for Big Data-driven Processes in Industry 4.0 describes the proposed architectural framework for big data processing in Industry 4.0. Section Application to Predictive Maintenance explains the application of the proposed framework in the context of predictive maintenance in the steel industry and presents the evaluation results. Section Conclusions and Future Work concludes the paper and presents our plans for future work.

## Literature Review

In this section, we present the literature review on the three pillars of the proposed architectural framework: Enterprise Integration and Interoperability (Section Enterprise Integration and Interoperability), big data processing (Section Big Data Processing), and Industry 4.0 (Section Industry 4.0). The literature review identifies the state-of-the-art in these research areas and enables the extraction of requirements for the design of the architectural framework for big data-driven processes in Industry 4.0.

### Enterprise Integration and Interoperability

Enterprise integration is the process of ensuring the interaction between enterprise entities necessary to achieve domain objectives (EN/ISO I9439, 2003), while enterprise interoperability refers to the ability of interactions (exchange of information and services) between enterprise systems (Chen et al., 2008). In this context, enterprise architecture facilitates enterprise modeling from various viewpoints and guides its implementation by providing a formal description of a system at a component level (ISO 15704, 2000; Open Group TOGAF, 2000; Bernus et al., 2003).

Since the 1980's, a lot of research has been conducted to develop enterprise architecture frameworks for enterprise integration, such as the Computer Integrated Manufacturing Open System Architecture (CIMOSA) (AMICE, 1993), the Purdue Enterprise- Reference Architecture (PERA) (Williams, 1994), the GIM architecture (Girard and Doumeingts, 2004), ARIS (Scheer, 1994), and Zachman Framework (Zachman, 1996). On top of them, the Generalized Enterprise-Reference Architecture and Methodology (GERAM) was developed (IFAC–IFIP Task Force, 1999), while additional frameworks, such as TOGAF (developed by Open Group on Architecture Framework) and C4ISR (or DoDAF) (DoDAF, 2007), as well as software engineering standards, were developed (e.g., ISO 15704, EN/ISO I9439, ISO 42010). Despite their differences, these architectures converge in three main levels of integration (Romero and Vernadat, 2016): (i) Physical Integration, which deals with systems interconnections and data exchange; (ii) Application Integration, which deals with interoperability of software applications and database systems; and (iii) Business Integration, which deals with co-ordination of functions, processes, and people.

On the other hand, several enterprise interoperability frameworks have been proposed in the literature (Chen et al., 2008), such as LISI (Levels of Information Systems Interoperability) (C4ISR, 1998), IDEAS interoperability framework (IDEAS, 2002), ATHENA interoperability framework (AIF) (ATHENA, 2003), Framework for Enterprise Interoperability (FEI), Big Data Value (BDV) Reference Model (BDVA, 2017), National Institute of Standards and Technology (NIST), and Big Data Interoperability Framework (NIST, 2019). In addition, during the last years, new domain-specific interoperability frameworks have been proposed, such as the European Interoperability Framework (EIF) (EIF, 2017), the Internet of Things-based interoperability framework for fleet management (Backman et al., 2016), the Smart City Interoperability Framework (Ahn et al., 2016), the interoperability framework for software as service systems in cloud (Rezaei et al., 2014), the International Image Interoperability Framework (Snydman et al., 2015), and the conceptual interoperability framework for large-scale systems (Selway et al., 2017). Overall, the enterprise interoperability frameworks can be seen in the frame of three main layers (Romero and Vernadat, 2016; Leal et al., 2020; Technical, Semantic, and Organizational).

### Big Data Processing

Big data is characterized by the 4Vs: Volume, Velocity, Variety, and Veracity (De Mauro et al., 2016). Volume is related to how much data is generated, velocity is related to how fast data is generated, variety is related to how many different types of data are generated, and veracity is related to how accurate data are (Chen et al., 2014; Xu and Duan, 2019). Big data analytics is classified in three main stages (Lepenioti et al., 2020): (i) descriptive analytics, identifying what has happened, examining why it happened, as well as providing real-time information about what is happening; (ii) predictive analytics, predicting what will happen and why; and (iii) prescriptive analytics, supporting decisions about what should be done and why.

The increasing size of big data poses challenges related to the complexity of big data-driven information systems and asks for novel techniques of software engineering for their design, analysis, and development (Varghese and Buyya, 2018; Xu and Duan, 2019; Davoudian and Liu, 2020). To this end, the literature has proposed asynchronous communications protocols, such as the Advanced Message Queuing Protocol (AMQP) (Vinoski, 2006) and the Message Queuing Telemetry Transport (MQTT) (MQTT, 2019), as well as scalable architectures, such as Lambda and Kappa Architecture (Davoudian and Liu, 2020). Moreover, the edge computing paradigm has emerged, aiming at addressing networking and computing challenges that cannot be met by existing cloud computing infrastructure (Trinks and Felden, 2018; Varghese and Buyya, 2018). Further, the fog computing aims at leveraging the existing computing resources on edge nodes or integrating additional computing capability between user devices and cloud data centers (Varghese and Buyya, 2018; Papageorgiou et al., 2019).

Apart from these, the recent emergence of a wide range of overlapping software frameworks in the literature, each one having a different focus, has resulted in the design of *ad-hoc* and complex architectural big data solutions (Davoudian and Liu, 2020). According to their focus and contribution, these research works can be classified into four categories: (i) empirically-grounded architectural design (Galster and Avgeriou, 2011; Angelov et al., 2012, Maier et al., 2013; Pääkkönen and Pakkala, 2015); (ii) implementation and deployment of big data systems (Schmidt and Möhring, 2013; Zimmermann et al., 2013; Salma et al., 2017); (iii) database management (Doshi et al., 2013; Zhong et al., 2013); and (iv) analytics integration (Westerlund et al., 2014; Sang et al., 2017). It should be noted that the literature is rich on domain-specific big data architectures, developed in order to address particular problems for specific application domains. For more details on the literature about software architectures, the reader may refer to Marjani et al. (2017) and to Davoudian and Liu (2020).

### Industry 4.0

#### RAMI 4.0

**Figure 1:**
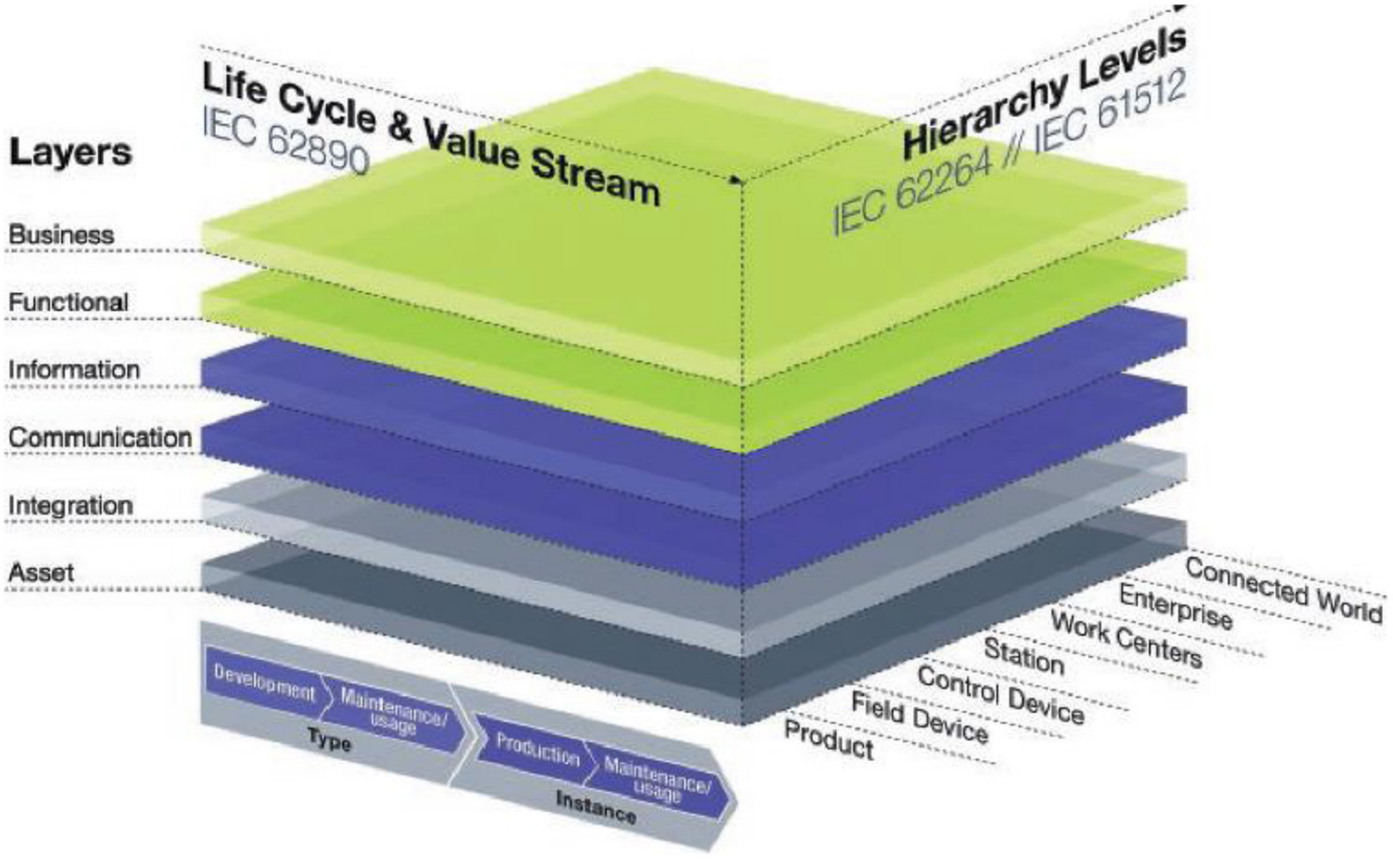
The RAMI 4.0 (Source: Hankel and Rexroth, 2015).

The German Federal Ministry of Education and Research defines Industry 4.0 as “the flexibility that exists in value-creating networks by the application of Cyber Physical Systems (CPS)” (Platform Industrie 4.0, 2019). In this context, Reference Architectural Model Industrie 4.0 (RAMI 4.0) is based on a three-dimensional model consisting of the Architecture Layers, Life Cycle and Value Stream, and Hierarchy Levels dimensions, as shown in Figure 1. RAMI 4.0 considers any technical asset of the factory as an entity that can be represented in the digital world to conform an I4.0 component. Industry 4.0-related core topics are on the way to being standardized with a strong focus on interoperability in order to ensure networking across company and industry boundaries (Deutsches Institut für Normung, 2019; Standardization Council Industrie 4.0, 2020).

The main scope of each dimension is described below. For a more detailed introduction to RAMI 4.0, the reader may refer to Hankel and Rexroth (2015), Adolphs et al. (2015), and Deutsches Institut für Normung (2019).

**Architecture Layers**: The Architecture Layers enable the development of Industry 4.0 software solutions in a consistent way so that different and mutually dependent manufacturing operations are interconnected, taking into account the physical and the digital world. RAMI 4.0 consists of six layers: Asset layer, Integration layer, Communication layer, Information layer, Functional layer, and Business layer.

**Life Cycle and Value Stream**: The second axis in RAMI 4.0 represents the lifecycle of products and systems and is taken from the IEC 62890 standard (International Electrotechnical Commission, 2017). The product lifecycle model introduces a differentiation between product type and product instance.

**Hierarchy Levels:** The third axis of RAMI 4.0 is the hierarchical representation of the different functional levels of the factory, based on the IEC 62264 (International Electrotechnical Commission, 2016) and IEC 61512 standards. These hierarchy levels are: Connected World, Enterprise, Site, Area, Work Centers, Work Units or Station, Control Device, Field Device, and Product.

In this context, a digital twin is the container for integrating information, executing operations, and producing data describing its activity which can be in different formats, from different software tools, and not necessarily deployed in one central repository (Ganz, 2018; Catarci et al., 2019). Both the physical and the digital twins are equipped with networking devices to guarantee a seamless connection and a continuous data exchange between a generic physical system (or process) and its respective Digital Twin (Platform Industrie 4.0, 2019), while they facilitate predictions about future situations and prescriptions about production optimization (Grieves and Vickers, 2017; Zillner et al., 2018; Barricelli et al., 2019). The digital twin is implemented by the Asset Administration Shell (AAS). The AAS consists of a number of sub-models in which all the information and functionalities of a given asset—including its features, characteristics, properties, status, parameters, measurement data, and capabilities—are described (Bedenbender et al., 2017). The German Federal Ministry of Economic Affairs and Energy provides specifications for the exchange of information with the AAS (German Federal Ministry of Affairs and Energy, 2018; German Federal Ministry of Economic Affairs and Energy, 2018).

#### Other Architectural Frameworks

Although, arguably, the most widespread and influential architectural framework in the manufacturing domain is RAMI 4.0, several other collaborative paradigms have emerged. The Industrial Internet Reference Architecture (IIRA), developed by the Industrial Internet Consortium (IIC) Task group, deals with different Industrial Internet of Things (IIoT) application domains such as Energy, Healthcare, Manufacturing, Public Domain, and Transportation (Industrial Internet Consortium, 2017a). An alignment of IIRA and RAMI 4.0 has been recently developed in order to identify the complementary, contradictory, and similar aspects of these two architectural paradigms (Industrial Internet Consortium, 2017b). On top of this, BDVA presented the big data challenges in smart manufacturing and designed the BDVA SRIA 4.0, a Reference Model for data-driven mapping of BDVA Reference Model to manufacturing scenarios, taking into account RAMI 4.0 and IIRA (BDVA, 2017). Other initiatives include NIST smart manufacturing (American National Institute of Standards Technology, 2017), China's National Intelligent Manufacturing System Architecture (IMSA) (Wei et al., 2017), Made in China 2025 vision for intelligent manufacturing, and (Ministry of Industry Information Technology of China Standardization Administration of China, 2015).

In parallel, the scientific literature has been contributing to the challenges of enterprise integration and interoperability in the smart manufacturing era (Zeid et al., 2019). Existing literature mainly focuses on the development of architectures for addressing lower level interoperability challenges of Industry 4.0, such as distributed storage, data aggregation, and service orchestration (Pisching et al., 2018; Bicocchi et al., 2019; Fraile et al., 2019) as well as big data infrastructures (Pedone and Mezgár, 2018; Calabrese et al., 2020). A considerable amount of research has also focused on architectures for CPS, digital twins, and AAS (Lee et al., 2015; Bader and Maleshkova, 2019; Bousdekis et al., 2020a; Cavalieri and Salafia, 2020). For more details, the reader may refer to Moghaddam et al. (2018), Cheng et al. (2018), Fraile et al. (2019), and Zeid et al. (2019).

## The Proposed Architectural Framework for Big Data-Driven Processes in Industry 4.0

In this section, we present the proposed architectural framework for big data-driven processes in Industry 4.0. First, we present the requirements to be fulfilled by the proposed framework (Section Requirements for the Architectural Framework). Second, we place the big data technologies and functions in the frame of RAMI 4.0 in order to assure enterprise integration and interoperability (Section Big Data Technologies and Functions in RAMI 4.0). On this basis, we provide a technical view of the 3-tier architecture in accordance with the Industry 4.0 principles and the existing big data technologies and architectures (Section Technical View of the Architecture).

### Requirements for the Architectural Framework

The literature review of Section Literature Review presented the background, as well as the main trends and challenges for each pillar of the proposed framework, i.e., Enterprise Integration and Interoperability, Big Data Processing, and Industry 4.0. Based on this analysis, we synthesized the requirements for each pillar. We concluded with 11 requirements, presented in Table 1. These requirements guide the design of the proposed architectural framework for enterprise integration and interoperability for big data-driven processes in the frame of Industry 4.0.

**Table 1:** The requirements for the architectural framework.

**Enterprise integration and interoperability**	
R1	The architecture shall support physical, application, and business integration.
R2	The architecture shall support technical, semantic, and organizational interoperability.
**Big data processing**	
R3	The architecture shall support the data analytics lifecycle, i.e., descriptive, predictive, and prescriptive analytics.
R4	The architecture shall support both stream processing and batch processing.
R5	The architecture shall support the whole cloud continuum, i.e., edge, fog, and cloud computing.
R6	The architecture shall integrate heterogeneous data sources.
**Industry 4.0**	
R7	The architecture shall conform to RAMI 4.0.
R8	The architecture shall tackle the uncertain manufacturing environment.
R9	The architecture shall be compatible with digital twins.
R10	The architecture shall provide various levels of insights.
R11	The architecture shall be generic and scalable.

### Big Data Technologies and Functions in RAMI 4.0

#### Architecture Layers

**Figure 2:**
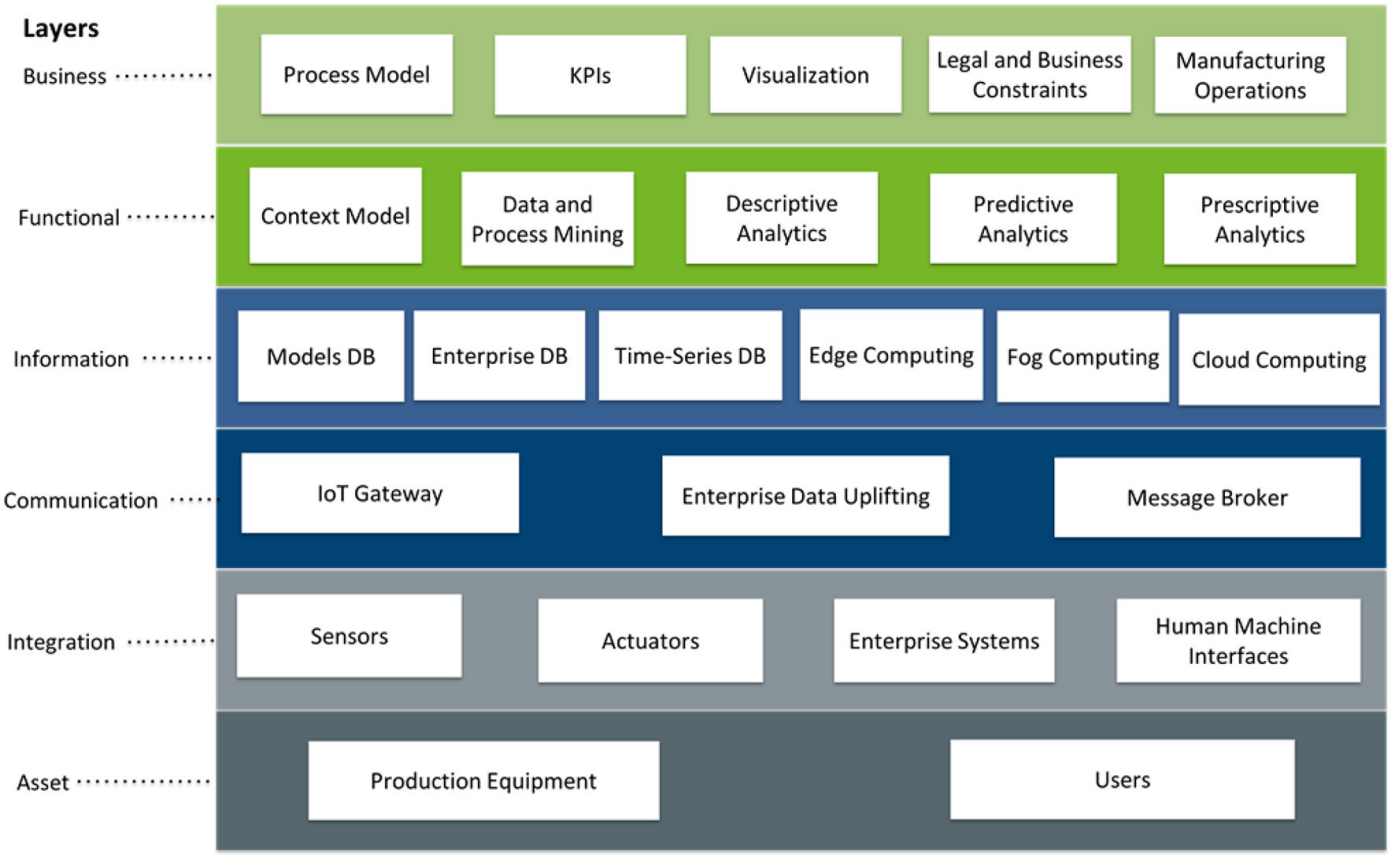
Big data technologies and functions for manufacturing operations in the frame of RAMI 4.0 Architecture Layers.

The proposed framework places the key components of a solution for big data-driven processes in the context of the RAMI 4.0 Architecture Layers, as depicted in Figure 2.

**Asset Layer:** Since this layer represents the reality, *Production Equipment* and *Users* are part of it. The Assets have their digital twin which is implemented with the AAS. The AAS can be applied on the level of a field device, a control device, and a station device. Moreover, the user, i.e., the Operator 4.0, can also have their “digital replica” according to the concept of “Human Digital Twin” (Bousdekis et al., 2020a).

**Integration Layer**: This layer provides information related to the assets in the appropriate format by connecting elements and people with information systems. This layer involves the *Sensors* and *Actuators* assigned to the machines as well as the *Enterprise Systems* (MES, ERP, etc.). It also includes the *Human Machine Interfaces* through which the users interact with the platform and the enterprise systems.

**Communication Layer**: Since this layer provides standardization of communication by means of uniform data format and deals with the physical support of information processing, it includes the *IoT Gateway*, the *Enterprise Data Uplifting*, and the *Message Broker*. The latter follows the AMQP or the MQTT protocol, while sensory and enterprise data are gathered with domain-specific data adapters. The machine-to-machine communication is implemented with the OPC UA protocol. In this way, data from various sources are collected for further processing in the Information Layer.

**Information Layer**: This layer provides pre-processing of events and execution of event-related rules by enabling their formal description for the interpretation of the information (Bousdekis et al., 2019a). It also manages data persistence and ensures consistent data integrity and transformation for feeding into the Functional Layer. Therefore, it includes sensor and enterprise data pre-processing in the Edge, Fog, and Cloud computational environments. *Edge Computing* contributes to this data pre-processing on the edge layer by performing edge analytics. *Fog Computing* leverages the resources on edge nodes and integrates additional computing capability along the entire data path between user devices and the cloud. *Cloud Computing* provides the infrastructure for the implementation of a big data reference software architecture. The types of data that need to be processed include real-time sensor data, historical sensor data, enterprise data, and human knowledge. In this case, the Lambda architecture is selected because it is able to incorporate stream processing and batch processing. Although the single stream processing engine of the Kappa architecture simplifies the implementation in completely data-driven computational environments, Industry 4.0 dictates that the human is an integral part of the process. Therefore, a batch processing engine is needed in order to facilitate the human-machine collaboration by embedding the expert knowledge at configuration or as soon as it becomes available. Apart from the real-time sensor data which are processed in the stream processing engine, historical sensor data and enterprise data can be processed in both engines. The information layer also includes the *Models DB*, for the storage of the simulation, context, knowledge and analytics models, the *Enterprise DB*, for the storage of the enterprise data, and the *Time-Series DB*, for the real-time data generated by equipment-installed sensors. In this way, the required data is extracted and combined accordingly in order to be available to the functions of the Functional Layer.

**Functional Layer**: This layer enables the formal description of functions and creates the platform for horizontal integration of various functions (Bousdekis et al., 2019a). It includes the run time and modeling environment for services supporting the business processes and a run time environment for applications and technical functionalities. In order to assure scalability and efficiency, the functionalities are developed as web services, following the background of SOA or EDA. Based upon the data integrity of the previous layer, the following functions are included:

- *Context Model*: It includes the definition of the manufacturing system's elements, including the assets, causes, and effects along with appropriate reactive and proactive actions. It also aims at performing knowledge representation and reasoning and, thus, it forms the basis for the enrichment of data analytics algorithms. In the current proposal, the context model is foreseen to be implemented with Probabilistic Web Ontology Language (PR-OWL) (Carvalho et al., 2017), in order to model the Bayesian relationships among the various entities of the context model (Setiawan et al., 2019) and to achieve uncertainty representation and reasoning. The queries can be performed with RDF and SPARQL.
- *Data and Process Mining*: Data mining deals with information extraction and discovery of structures and patterns in large and complex data sets (Hand and Adams, 2014). Process mining extracts knowledge from event logs stored in the information systems in order to discover, monitor, and improve processes (Van Der Aalst et al., 2011). In the proposed approach, they facilitate the structuring and analysis of enterprise data and event logs in order to extract meaningful insights about the past performance of manufacturing business' processes and KPIs. They also enable longer-term decisions, while their results contribute to the enrichment and labeling of the descriptive, predictive, and prescriptive analytics outcomes.
- *Descriptive Analytics*: In a stream processing context, descriptive analytics deals with (deep) machine learning methods and algorithms for anomaly detection (Yue et al., 2019). In the proposed architecture, descriptive analytics is implemented as real-time algorithms on the basis of sensor-generated data streams in order to detect abnormal behaviors.
- *Predictive Analytics*: Predictive analytics are executed both offline and in real-time. It includes the development of offline predictive analytics models based on historical sensor data until a decision horizon enriched by the Data and Process Mining and the Context Model functions. Moreover, at runtime, when the descriptive analytics function detects an abnormal behavior, predictive analytics retrieves the appropriate offline model and predicts the future situation and/or the time of its occurrence.
- *Prescriptive Analytics*: Prescriptive analytics is triggered by real-time predictions about future situations in order to generate recommendations about proactive actions and their optimal time and formulate the action plan. The prescriptive analytics models have been developed offline by taking advantage of the Data and Process Mining function as well as the Context Model.

**Business Layer**: This layer ensures the integrity of functions in the value stream and enables mapping business models and the outcomes of the overall process (Bousdekis et al., 2019a). In other words, it assures the enterprise integration and interoperability by providing the interfaces with the rest of the business functions. It takes into account the constraints, rules, and policies that affect the system operation and facilitates the interaction of manufacturing operations with the overall business environment. To do this, it provides the *Process Model* in accordance with the business models and *KPIs* defined at the strategic level, also taking into account the *Legal and Business Constraints*. In this way, it provisions both the organizational and legal interoperability. Finally, this layer provides the business interface with other *Manufacturing Operations* of the business environment as well as with the associated users through *Visualization* of results in order to achieve explainability of the AI algorithms that were implemented in the Functional Layer and, thus, to be compatible with the “Ethics Guidelines for Trustworthy Artificial Intelligence” for the development of ethical and human-centric AI, as has been reported by the High-Level Expert Group on AI (European Commission, 2019).

#### Life Cycle and Value Stream

The proposed approach facilitates the interchange of data and data analytics outcomes to all the stakeholders of the supply chain and at all stages of the manufacturing operations. This fact enables timely and reliable cooperation among them throughout the whole lifecycle of operations.

#### Hierarchy Levels

**Figure 3:**
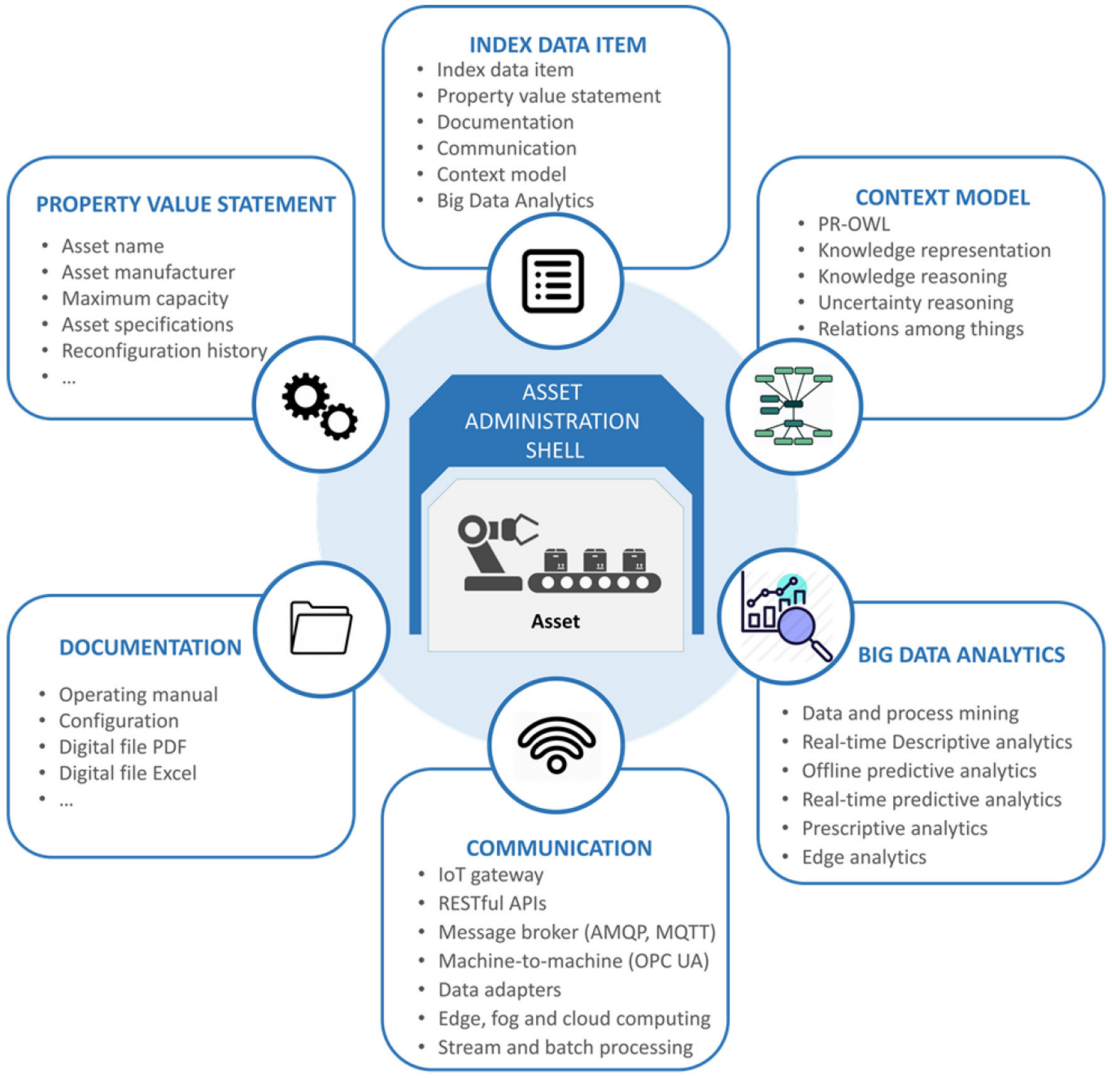
The I4.0 AAS of the big data-driven manufacturing operations' Digital Twins.

The proposed approach is also compatible with the Hierarchy Levels of RAMI 4.0. The I4.0 AAS concept enables its application to the various Hierarchy Levels on the basis of a defined Asset or as a synthetics AAS, combining the AAS of various Assets at various levels. To achieve this, the AAS, which implements the Digital Twin and ranges from the Asset Layer of RAMI 4.0 to the Functional Layer, is formulated as shown in Figure 3. It is based on the AAS template proposed by Ye and Hong (2019) and provides a generalized and data-driven way of constructing the associated sub-models. The AAS can be extended in order to include additional asset-specific sub-models (e.g., quality, maintenance, supply chain, etc.).

### Technical View of the Architecture

**Figure 4:**
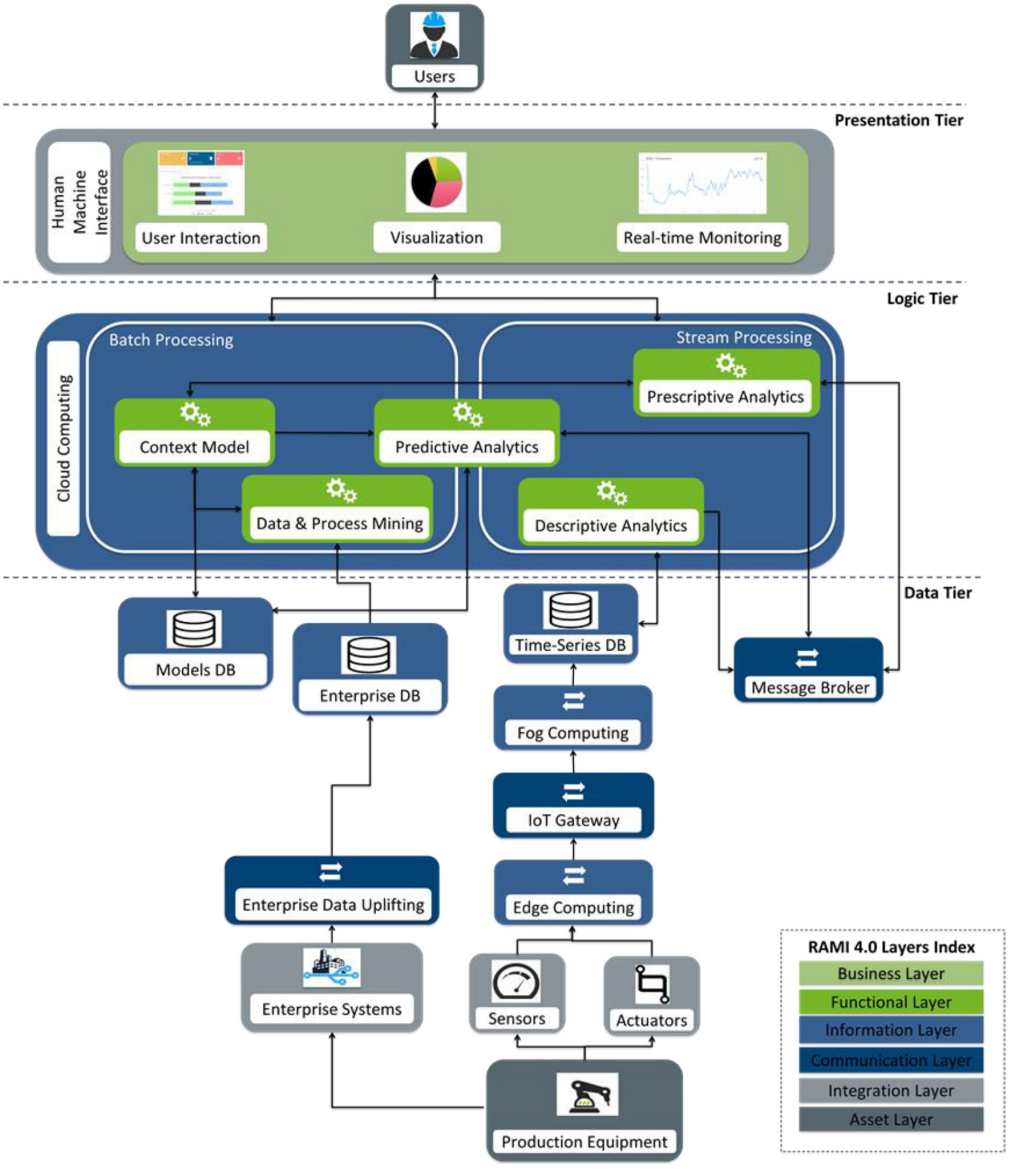
The 3-tier technical view of the architecture for big data-driven processes in Industry 4.0.

Based upon the aforementioned structuring of big data technologies and functions in the frame of RAMI 4.0, we design the technical view of the proposed architecture for big data-driven processes in Industry 4.0, depicted in Figure 4. It shows the main interactions and the data flow among the components through the definition of end-to-end integration and communication processes and consists of three tiers: Presentation Tier, Logic Tier, and Data Tier. The technical view of the architecture drives the development procedure of associated information systems and platforms in accordance with the Industry 4.0 principles.

Below, we explain the interactions and the data flow among the components of the aforementioned architecture based upon the descriptions provided in Section Big Data Technologies and Functions in RAMI 4.0. The Logic Tier is put at the core of the description, while its functions are presented in two steps according to whether the data flow takes place at design time or at runtime:

**Design time**: The entities of the *Context Model* are defined based on the expert knowledge that is inserted through the *User Interaction* of the Presentation Tier, while their Bayesian relationships are extracted by the *Data and Process Mining* function of the Logic Tier and are communicated through RESTful APIs. The *Data and Process Mining* function retrieves data in order to produce process models, to analyze past performance, and to estimate KPIs. This data are derived from *Enterprise Systems* and are stored in the *Enterprise DB* (a NoSQL DB) of the Data Tier through *Enterprise Data Uplifting*. Its outcomes are exposed to the user through *Visualization* of the Presentation Tier. The Context Model and the outcomes of Data and Process Mining are stored in the Models DB.

**Runtime**: The *Descriptive Analytics* function processes sensor-generated data streams in real-time in order to detect the actual performance of the *Production Equipment*. To do this, it mainly implements (deep) machine learning algorithms. This data are stored to the *Time-Series DB* and are communicated with the AMQP and the MQTT protocols. An initial pre-processing of the data may have taken place through edge analytics techniques in *Edge Computing*. These outcomes have been extracted through an *IoT Gateway*, while they have been further processed in *Fog Computing*. The *Predictive Analytics* function relies on both *Batch Processing* and *Stream Processing*. At Stream Processing, it receives streams of the Descriptive Analytics outcomes through the *Message Broker* and generates predictions about the future states of the *Production Equipment*. The predictions are generated according to the predictive analytics models that have been developed at Batch Processing based on historical sensor data enriched by the *Data and Process Mining* and the *Context Model* functions. The *Prescriptive Analytics* function receives streams of predictions through the *Message Broker* and generates prescriptions about proactive actions, i.e., actions that mitigate the impact of a future undesired event or exploit future opportunities. The Prescriptive Analytics models are enhanced by the *Data & Process Mining* and the *Context Model* functions. The outcomes of Descriptive, Predictive, and Prescriptive Analytics are exposed to the user through *Real-time Monitoring* of the Presentation Tier, while they are stored to the *Models DB* of the Data Tier.

## Application to Predictive Maintenance

In this section, we present the instantiation of the proposed architectural framework to predictive maintenance and the deployment of an associated platform to a steel industry case study. More specifically, we describe the motivation for selecting the maintenance process (Section Predictive Maintenance in Industry 4.0), we instantiate the proposed framework to the predictive maintenance context (Section Instantiation of the Proposed Architectural Framework to Predictive Maintenance), and we present the deployment of a predictive maintenance platform, developed according to the proposed framework, to a case study from the steel industry (Section Case Study in the Steel Industry).

### Predictive Maintenance in Industry 4.0

Predictive maintenance is an indispensable aspect of Industry 4.0, since it aims at achieving availability of the production equipment while avoiding unplanned downtimes with the use of condition monitoring. Predictive Maintenance in the context of Industry 4.0 is the maintenance strategy that takes advantage of the huge amounts of real-time and historical data in the enterprise ecosystem in order to detect early anomalies in equipment behaviors, to predict the future health state of the equipment and potential future failure modes, and to formulate proactive maintenance plans with the aim to eliminate or mitigate the impact of the predicted failures (Bousdekis et al., 2020b). Due to its importance, the German Federal Ministry of Affairs and Energy has published “The Standardization Roadmap of Predictive Maintenance for Sino-German Industrie 4.0/ Intelligent Manufacturing” (German Federal Ministry of Affairs and Energy, 2018), while the European Federation of National Maintenance Societies (EFNMS) has published the maintenance Body of Knowledge (BoK) (EFNMS, 2019).

**Figure 5:**
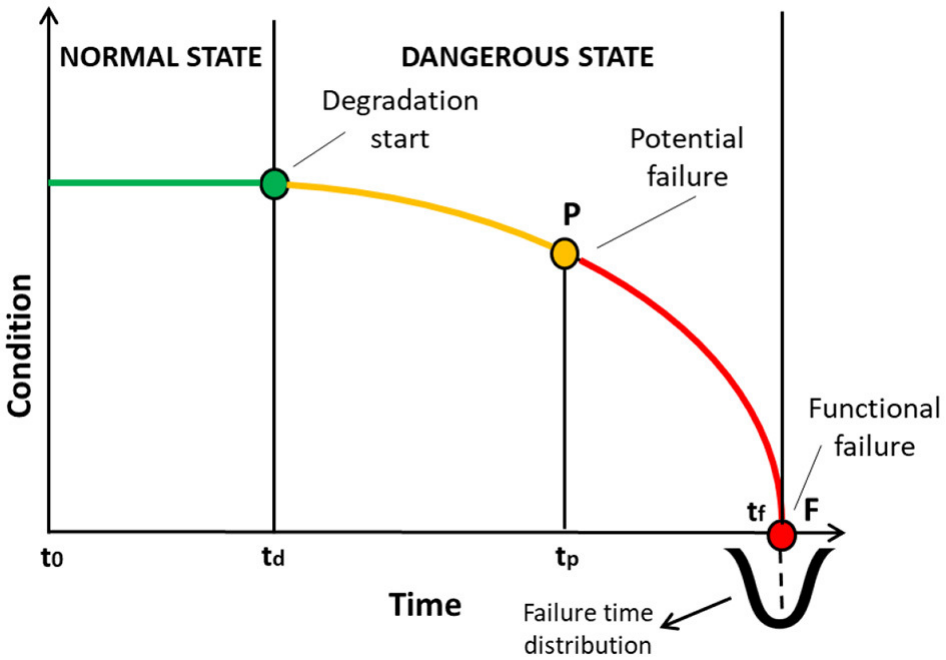
Predictive maintenance in the context of the P-F curve.

**Figure 6:**
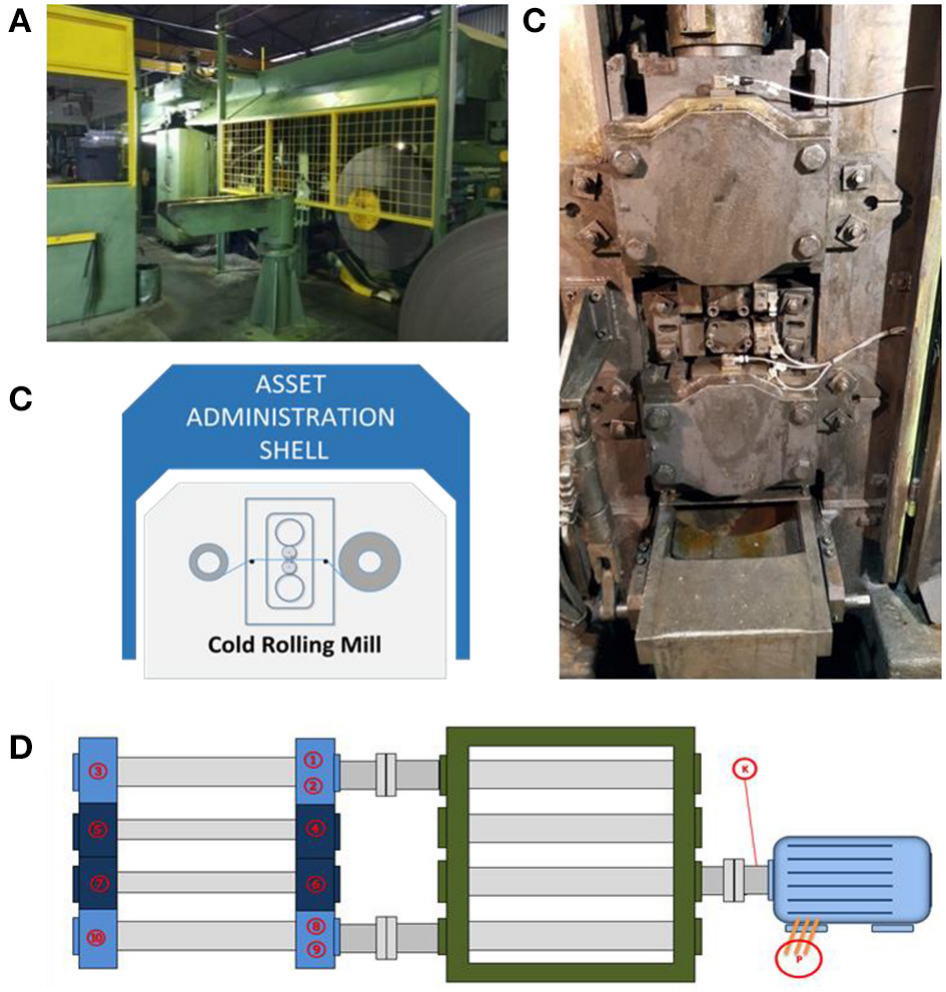
The milling station: **(A)** an overview; **(B)** a representation of the asset in the AAS; **(C)** the rolls when the main casing is open and the placement of sensors; **(D)** the sensors placement in the infrastructure setup.

Predictive maintenance has gathered increasing interest in both literature and practice. However, the lack of successful case studies and the development of *ad-hoc* approaches and platforms have led the manufacturers to be reluctant for its adoption (Guillén et al., 2016; Hribernik et al., 2018). The potential of predictive maintenance can be demonstrated through the concept of P-F curve, as shown in Figure 5. The P-F curve is a well-established representation of asset's behavior. According to the P-F curve, the condition of an asset deteriorates over time, leading to functional failure. Therefore, the failure is considered as a process instead of an instant event. As shown in Figure 6, this approach provides an opportunity time window, i.e., the P-F interval, between the time of the potential failure (P), i.e., the point that it can be found out that the equipment is failing, and the functional failure (F), i.e., the point when the equipment actually fails, within which proactive decisions and actions can be taken. The point F is typically a distribution of the possible failure times for the failure mode under examination, derived from the historical data analysis. While Breakdown Maintenance is implemented after the point F and Time-Based Maintenance is scheduled at specific time intervals in order to avoid F, predictive maintenance can take advantage of the big data in order to maximize the P-F interval closer to the degradation start and support timely and cost-efficient decisions even before the symptoms are visible by humans.

### Instantiation of the Proposed Architectural Framework to Predictive Maintenance

The systematic representation of a predictive maintenance solution enables the reusability and knowledge transfer, an aspect of outmost importance in Industry 4.0 platforms (Bousdekis et al., 2018a; Gröger, 2018). The proposed approach provides the capability of designing the “Predictive Maintenance Digital Twin” in order to facilitate the development of a predictive maintenance platform in the frame of Industry 4.0. Below, we illustrate how the functions of the Functional Layer are instantiated to predictive maintenance.

- *Context Model*: It includes the definition of the manufacturing system including the assets, failure causes, failure modes, and effects, along with appropriate reactive and proactive actions in order to create the maintenance data model. Existing related literature usually develops data models and ontologies based on the FMECA background (Zhou et al., 2015; Guillén et al., 2016; Nunez and Borsato, 2017; Ali and Hong, 2018; Hribernik et al., 2018). However, their deterministic and static nature creates obstacles to the full exploitation of big data in the frame of Industry 4.0 (Bader and Maleshkova, 2019). The PR-OWL can enable the representation of domain knowledge enhanced by data analytics in the form of uncertain relationships between the FMECA entities, e.g., failure causes, the failure modes, and the mitigating actions, while the root causes are mapped to the available sensors that serve as indirect indicators of the failure modes.
- *Data and Process Mining*: It extracts useful insights about root causes of failures and maintenance-related business processes based upon the enterprise and operational data [e.g., Overall Equipment Effectiveness (OEE) data, Statistical Process Control (SPC), Enterprise Resource Planning (ERP), and Computerized Maintenance Management System (CMMS), maintenance event logs] in order to construct the maintenance process model. Data mining algorithms have been widely used in the related literature (Accorsi et al., 2017), while process mining is an emerging research area (dos Santos Garcia et al., 2019).
- *Descriptive Analytics*: In a predictive maintenance context, descriptive analytics is implemented as real-time diagnostic algorithms. Diagnosis aims at assessing the actual health state of the equipment and identifying abnormal behaviors based on sensor-monitored indicators of degradation, e.g., when anomalies are detected (Bousdekis et al., 2015). The literature is rich on real-time diagnostic algorithms; see e.g., Xu et al. (2017) and Li et al. (2020).
- *Predictive Analytics*: Predictive analytics is implemented as prognostic algorithms, triggered by real-time diagnostic outputs. Prognosis aims at predicting the future health state of the equipment, particularly when a failure mode is expected to occur, and estimating the Remaining Useful Life (RUL) (Bousdekis et al., 2015). This function includes the development of offline predictive analytics models based on historical sensor data that indicate the degradation process until the failure. In this sense, the models utilize information that exists in maintenance logs and in the context model, such as the time that a failure occurred, the type of the failure mode, the asset, etc. At runtime, when the descriptive analytics function detects an abnormal behavior which moves the equipment from a normal state to a deteriorating state, predictive analytics retrieves the appropriate offline model and predicts the failure mode and/or the time of the failure occurrence. Prognostic algorithms have been widely investigated in the last years; see e.g., Zonta et al. (2020).
- *Prescriptive Analytics*: Prescriptive analytics models are triggered by real-time predictions about future failure modes in order to generate recommendations about proactive actions and formulate the maintenance plan. The prescriptive analytics models have been developed offline by taking advantage of the Data and Process Mining function as well as the Context Model. Although the development of maintenance decision making algorithms is a well-established area (Ruschel et al., 2017), the sensor-driven computational environment and the need for proactive actions ahead of time (instead of reacting to incoming events) ask for novel proactive decision making methods (Bousdekis et al., 2019b).

Table 2 provides an overview of the instantiation of the proposed architecture to predictive maintenance by addressing the Functional Layer of RAMI 4.0. More specifically, it defines the five big data functions of the proposed architecture in the frame of predictive maintenance along with related categories of methods and their inputs and outputs. Moreover, it defined the data processing approach, the data storage high-level specifications, and the appropriate communication protocols for each function.

**Table 2:** Instantiation of RAMI 4.0 functional layer to predictive maintenance.

**Function**	**Context model**	**Data and process mining**	**Descriptive analytics**	**Predictive analytics**	**Prescriptive analytics**
Predictive maintenance	Maintenance data model	Maintenance process model	Diagnosis	Prognosis	Maintenance planning
Methods	Probabilistic ontology, belief networks, uncertainty reasoning	Knowledge discovery, statistical analysis, descriptive, predictive	Signal processing, unsupervised machine learning, deep learning	Unsupervised and supervised machine learning, deep learning	Supervised and reinforcement learning, operational research
Inputs	Domain knowledge Enterprise data Analytics models Process models	Enterprise data Maintenance logs	Sensor data	Current health state Historical prognostic models Context	Estimated RUL Failure PDF Context Human feedback
Outputs	System definition Uncertain relationships Contextual elements	KPIs Business performance Process models	Time and frequency features Current health state Early warnings	Estimated RUL Break down prediction Failure PDF	Proactive actions Maintenance plan Optimal time of maintenance
Data processing	Batch processing	Batch processing	Stream processing	Stream processing (batch processing)	Stream processing
Data storage	OWL 2 RL profile Triplestore NoSQL	Time-series DB OWL 2 RL profile NoSQL	Time-series DB	Time-Series DB OWL 2 RL profile NoSQL	Time-series DB OWL 2 RL profile NoSQL
Communication protocol	RESTful APIs	RESTful APIs	AMQP	AMQP RESTful APIs	AMQP RESTful APIs

### Case Study in the Steel Industry

In this section, we demonstrate a predictive maintenance platform that was developed in accordance with the proposed architectural framework for big data-driven processes in Industry 4.0 and based upon its instantiation to predictive maintenance, as presented in Section Instantiation of the Proposed Architectural Framework to Predictive Maintenance. It has been applied in three use cases from different manufacturing sectors: the steel industry, domestic appliances production, and the aerospace industry. The following demonstration deals with the steel industry.

#### The Manufacturing Process and Equipment

The case under examination is the cold rolling process. Cold rolling is a manufacturing process of metal deformation involving a pair of rotating metal rolls aiming at reducing the cross-sectional area or shaping a metal piece below its recrystallization temperature. The main components of the milling station are:

- The work rolls: a pair of rolls responsible for the actual milling until the required width is achieved.
- The backup rolls: a pair of rolls transmitting motion to the working rolls.
- The motor unit: the component supporting the rotation of the backup rolls.

Figure 6A shows the milling station on the shop floor; Figure 6B illustrates the cold rolling manufacturing process. Figure 6C depicts the work and the backup rolls. During the operation, the rolls are continuously being sprayed by soap oil in order to reduce heat and friction. Figure 6D depicts the infrastructure setup for sensor data acquisition and the placement of the accelerometers. Their description is presented in Table 3.

**Table 3:** The installed accelerometers.

**Sensor ID**	**Measurement point**	**Sensor direction**
1	Upper backup roll – DE side	Vertical
2	Upper backup roll – DE side	Axial
3	Upper backup roll – NDE side	Vertical
4	Upper working roll – DE side	Reverse horizontal
5	Upper working roll – NDE side	Horizontal
6	Down working roll – DE side	Reverse horizontal
7	Down working roll – NDE side	Horizontal
8	Down backup roll – DE side	Vertical
9	Down backup roll – DE side	Axial
10	Down backup roll – NDE side	Vertical

Before the installation of sensors, maintenance was performed on a time-based mode. The rolls were replaced every 8 h (i.e., when there was a shift change) regardless of their health state. According to their condition, identified with visual inspection, the removed rolls were subject either to repair or they were sent to waste. In this way, on the one hand, replacement took place even if it was not necessary and, on the other hand, unexpected failures occurred between successive replacements. This fact led to high maintenance costs, despite the improvements of the last years due to the adoption of a Total Productive Maintenance (TPM) management program.

#### The Datasets

The data used in this case were derived from sensors and a CMMS, while the required expert knowledge was embedded through a Graphical User Interface (GUI). The sensor infrastructure consists of 10 Accelerometers collecting vibration data which are gathered in an MVX which are then transmitted *via* Modbus TCP to a Siemens S7-1500 PLC. The values are exposed from the PLC to the DB port and can thus be collected *via* external modules that have access to the PLC *via* the network. An adapter was developed in order to sample the DB Port every 5 ms – 5 s. The data are then processed *via* a Storm-Kafka pipeline and are stored and retrieved into the *Time-series D*. This pipeline is responsible for performing normalization procedures before the data are pushed to the Logic Tier. Normalization is also configurable and can be adjusted by attaching new Storm Bolts. A set of Bolts for rounding continuous variables has been deployed. The summary of the streaming dataset derived from the aforementioned accelerometers is presented in Table 4. In addition, there is a CMMS which stores operational and enterprise data and performs basic calculations about OEE, time-based maintenance plan, failure modes, downtime, etc. The summary of the CMMS dataset used in this case is presented in Table 5.

**Table 4:** Summary of the streaming dataset.

Dataset title	Roller vibrations
Origin	Milling station
Sensor type	Accelerometer
Physical world measurements	Acceleration, velocity, shock, and overall bearing
Sensor reporting frequency	10 readings per minute (configurable)
Data stream rate	~8 kb per minute
Sensor input signal(s)	Mechanical
Data type	Acceleration, velocity, bearing: Float Shock: Integer
Interfaces to obtain sensor readings	PLC TCP connection

**Table 5:** The CMMS dataset.

Dataset title	Operational and legacy
Data type	**Roll data** Roll position: upper roll/down roll Roll ID Roll past operation Input characteristics: timestamp, roll diameter Output characteristics: timestamp, reason of replacement (type of failure mode/planned maintenance) **Production data** KPIs: number of failures, time of planned maintenance, OEE, MTBF, MTTR
Interface	Data uplifting or API

#### Implementation of the Architecture

The core technology stack of the predictive maintenance platform which implements the 3-tier architecture for big data-driven processes in Industry 4.0 is shown in Table 6. The platform embeds various algorithms for each function, as shown in Table 7. Some of these algorithms have been developed within the platform while others are retrieved through APIs from open source data analytics tools. In this way, the platform is able to tackle a wide variety of cases, assets, and degradation behaviors, while it is extensible in order to embed more algorithms.

**Table 6:** Technology stack.

**Presentation tier**	
User interaction	Thymeleaf
Visualization	Grafana, Kibana, Thymeleaf
Real-time monitoring	Grafana, Kibana, Thymeleaf
**Logic tier**	
Context model	Spring Boot framework, Apache Maven
Data and process mining	Django Web Framework, django-rest-framework, Elasticsearch, Jupyter, Pandas, scikit-learn, numPy, somPy, Keras, pm4Py
Descriptive analytics	Spring Boot framework, Apache Commons, Netlib, Common Math, MOA: Massive Online Analysis, MathParser Org MXparser, Elasticsearch
Predictive analytics	Spring Boot framework, Apache Commons, Weka, Common Math, MOA: Massive Online Analysis, Netlib
Prescriptive analytics	Apache Maven, Spring Boot framework, Drools, BURLAP, Common Math, Netlib
**Data tier**	
Models DB	MongoDB, MySQL, MariaDB
Enterprise DB	MongoDB, PostgreSQL
Time-series DB	InfluxDB
Message broker	Apache Kafka

**Table 7:** The algorithms implemented in the platform.

Data and process mining	Linear Regression, Bayesian Networks, Self-Organizing Map (SOM), K-means clustering, Support Vector Machines (SVM), Decision Tree (DT), Random Forest (RF), Inductive Miner, Fuzzy Miner
Descriptive analytics	Feature Extraction, k-Nearest Neighbor, association rules, online Bayesian changepoint detection
Predictive analytics	Logistic Regression, Exponential fitting, Weibull fitting, Hidden Markov Model (HMM)
Prescriptive analytics	Association rules, Bayesian Networks, Markov Decision Process, Reinforcement Learning

At design time, the *Context Model* represents and stores probabilistic relationships among failure causes, failure modes, and mitigating actions. It is in accordance with MIMOSA OSA-CBM data model, while it introduces the capability of uncertain relationships among the entities according to PR-OWL. The *Data and Process Mining* function creates the maintenance process model and enriches the offline predictive analytics models. Moreover, it feeds into the context model with the cost models of the failure modes and the mitigating actions. At runtime, the platform provides real-time monitoring of the vibration and ensures that the gathered data at the on-site PLC are transmitted through the communication channel. The acquired data feed into the stream processing functionalities, i.e., Descriptive, Predictive, and Prescriptive Analytics, which implement various data fusion, (deep) machine learning, and optimization algorithms, which are configurable according to the specific use case requirements and complexity.

#### Illustrative Scenario

**Figure 7:**
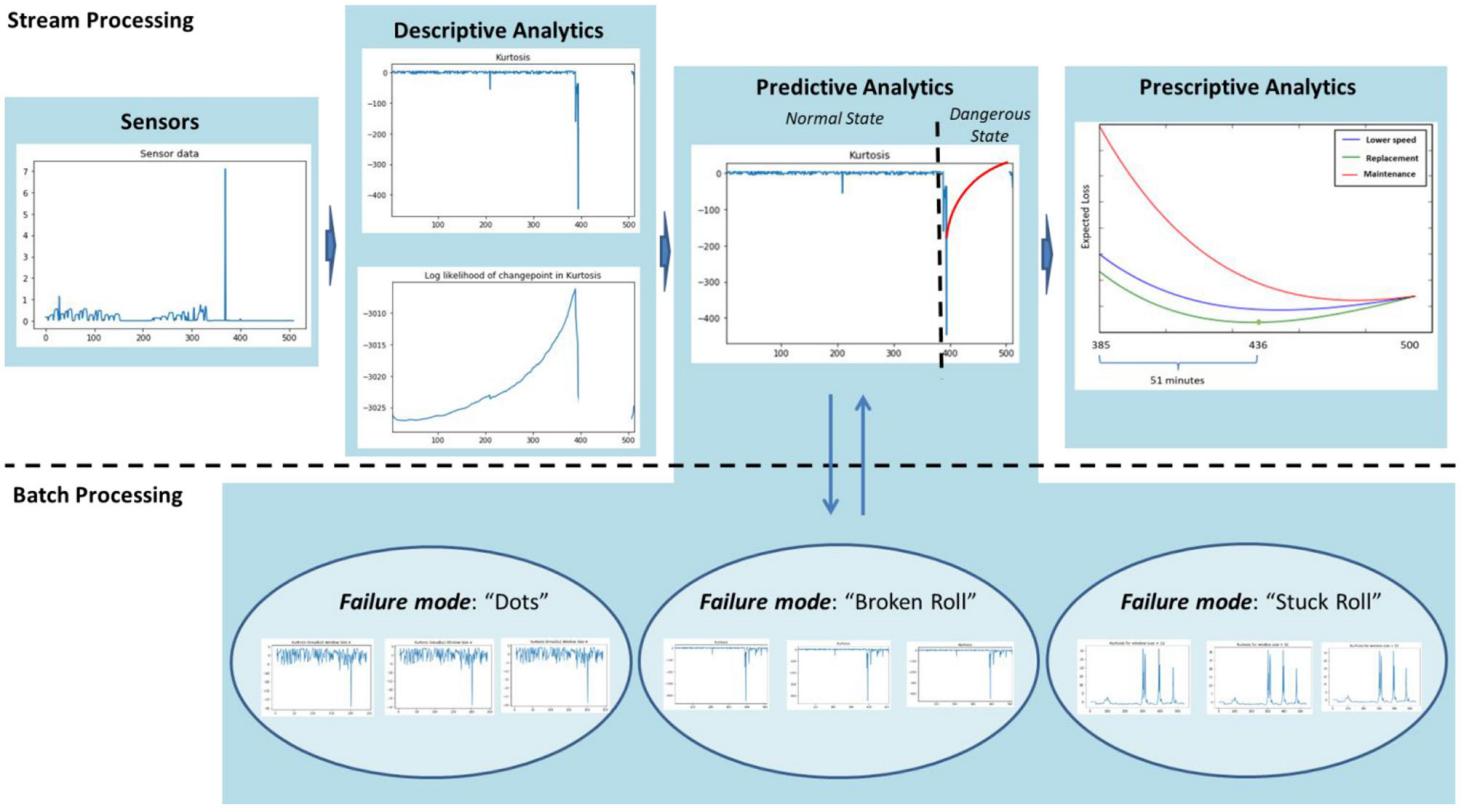
An illustrative scenario in the context of the case study.

As already mentioned, the platform embeds various algorithms in order to deal with the complexity and the requirements of the complex manufacturing environment, e.g., various assets, different degradation behaviors, different specifications, etc. In order to demonstrate a typical information flow across the aforementioned functions, we herein present an instance of the platform in the case study as an illustrative scenario. The scenario in the case study under examination is depicted in Figure 7.

In this scenario, *Descriptive Analytics* performs feature extraction with rolling kurtosis and online Bayesian changepoint detection (Wen et al., 2018) in order to estimate in real-time the log likelihood of having a changepoint from the normal state to the dangerous state of equipment. In Figure 7, it identifies a changepoint 385 min after the setup of the machine. This outcome triggers the *Predictive Analytics* service in order to provide a prediction about the failure mode occurrence by retrieving the most similar cluster that corresponds to a failure mode and applying Weibull fitting (Zhang et al., 2018). Therefore, it predicts that the failure mode “Broken Roll” will occur in 115 min. This prediction feeds into *Prescriptive Analytics* which recommends the optimal proactive action, out of the alternative actions for this failure mode, along with the optimal time of its application. In the current scenario, the prescriptive analytics model has been developed as a Markov Decision Process (MDP) (Bousdekis et al., 2018b) and there are three alternative maintenance actions: lower the speed of the machine, replace the rolls, and perform full maintenance on the equipment. As shown in Figure 7, the optimal action is to replace the rolls in 51 min, because at that time, the expected loss is minimized.

#### Evaluation Results

We evaluated the value of the proposed approach by performing a before/after analysis of KPIs retrieved by the CMMS. The comparison was performed on the basis of 2 complete years. In the 1st year of the evaluation period (“before”), the company performed time-based maintenance by replacing the rolls every 8 h, according to the supplier's specifications. As a result, either the rolls were replaced in their normal state or unexpected breakdowns took place in this time interval. In the 2nd year, the aforementioned platform had been deployed and the company started performing predictive maintenance of the rolls. In this way, not only the decisions about the roll replacement were taken dynamically, but also it adopted imperfect maintenance actions (e.g., lower the speed of the mill, increase the soap oil to eliminate friction, optimal utilization of repaired rolls, etc.) that extend the lifetime of the equipment when downtime is not acceptable (e.g., when customers' demands need to be met).

The evaluation focused on the aforementioned milling station and not on the whole production process in order to eliminate other factors that may affect the KPIs' values. It is expected that the effect of the proposed approach can be multiplied if it is applied to the whole factory and for a longer period of time. The results are shown in Table 8. We also interviewed key persons from the company, such as the General Manager, the Quality Manager, the Production Manager, and the operators of this milling station. According to the results, the number of failures was decreased by 47.69%, the time for performing planned (time-based) maintenance was decreased by 62.5%, the OEE was increased by 5.03%, the Mean Time Between Failures (MTBF) was increased by 22.90%, and the Mean Time To Repair (MTTR) was decreased by 21.88%.

**Table 8:** Evaluation results of KPIs in the steel industry.

***KPI***	**Number of failures**	**Planned maintenance duration**	**OEE**	**MTBF**	**MTTR**
Before	96	2,340 min	59.43%	2,135 min	117 min
After	65	1,440 min	64.46%	2,772 min	96 min
Improvement	*47.69%*	*62.5%*	*5.03%*	*22.90%*	*21.88%*

#### Lessons Learned

In this section, we summarize the lessons learned from all three use cases (steel industry, domestic appliances production, and aerospace industry), mainly related to the enterprise integration and interoperability challenges. In this sense, we emphasize the challenges with respect to the complexity of applying the proposed framework and to the integration with the manufacturing environment, information systems, and measuring devices. Below, we discuss the main lessons learned.

**Combination of process knowledge and data analytics**: On a business level, collaboration and communication between domain experts and data analysts is a challenging task. On the one hand, the data analysts need to understand the manufacturing process, it potential and constraints, as well as the business requirements in order to decide on the functionalities, the algorithms, and the configuration of the platform to the specific business needs. On the other hand, the domain experts need to understand the system and technical requirements that may lead, not only to new investments on information systems and sensor infrastructure, but also to a disruptive way of thinking and practice.

**Project management**: The adoption of disruptive technologies in the frame of Industry 4.0 needs efficient project management. Such projects usually last for a long period of time in order to tackle the large variety of integration and data management challenges and to sufficiently assess the efficiency of the software solutions. Long-lasting software maintenance and support is also an important aspect.

**Data privacy and security:** The manufacturing data is highly confidential for the manufacturers, since they are critical to their processes. Moreover, the increasing use of sensors and actuators on the shop floor makes cybersecurity a topic of outmost importance for the robustness of operations and the safety of operators. For these reasons, manufacturing companies are usually reluctant to open-source platforms “intervening” with their legacy systems and sensor infrastructure and externally processing the data. Such platforms should use state-of-the-art technologies and mechanisms that ensure data security, while a close collaboration with the enterprise systems and sensors is essential.

**Utilization of heterogeneous data sources**: The manufacturing environment includes various and heterogeneous data sources that have the potential to provide insights on various aspects of the processes. This heterogeneity is caused by, among others, the co-existence of old-fashioned systems and disruptive Industry 4.0 technologies. Therefore, an Industry 4.0 platform needs to take advantage of all the data sources which may include sensors, actuators, legacy and operational systems, enterprise systems, and Excel files, but also expert knowledge. The identification of all the available data sources, the development of appropriate interfaces, and the implementation of the right algorithms are among the main challenges in the deployment of the proposed solution.

**Integration to the legacy and operational systems**: Legacy and operational systems are usually proprietary solutions, something which poses additional challenges to the integration of open-source platforms. Apart from the close collaboration with the provider, it is important to define the required data formats, and process the data in order to enrich the context model and the data analytics algorithms. In this sense, the database management of the proposed solution is crucial for the storage and retrieval according to the consuming functions. When this is not possible, legacy data uplifting can be applied.

**Integration to the sensor infrastructure**: Integration to the sensor infrastructure also requires a close collaboration with the provider in order to develop adapters capable of extracting the data at a pre-configured sampling time. The adapters need to be configured in order to sample the DB port at appropriate times according to the specific process (e.g., frequency of events, criticality of operations, time constraints, etc.). They should ensure data security.

## Conclusions and Future Work

Traditional manufacturing businesses lack the standards, skills, processes, and technologies to meet today's challenges of Industry 4.0 driven by an interconnected world. Enterprise Integration and Interoperability can ensure efficient communication among various services in alignment with the business needs and requirements. However, the data management challenges affect not only the technical implementation of software solutions but the function of the whole organization. A key issue in Industry 4.0 is the effective application of the Reference Architecture Model Industrie (RAMI) 4.0 in various manufacturing operations. In this paper, we bring together Enterprise Integration and Interoperability, Big Data Processing, and Industry 4.0 in order to identify synergies that have the potential to enable the so-called “Fourth Industrial Revolution.” On this basis, we propose an architectural framework for designing and modeling Industry 4.0 solutions for big data-driven manufacturing operations. We demonstrate the applicability of the proposed framework through its instantiation to predictive maintenance, a manufacturing function that increasingly concerns manufacturers due to the high costs, the safety issues, and the complexity of its application. The proposed approach achieved to exploit the full potential of predictive maintenance in a case study from the steel industry, since it provides a systematic way of designing the maintenance operations and developing a software platform. At the same time, the developed solution can be seen in the context of the whole enterprise architecture, according to the digital manufacturing strategy, in order to balance the wide-ranging—vertical and horizontal—effects within the organization. The effect of such a solution is strongly affected by the data availability (quality and quantity) and the algorithms suitability; however, the enterprise integration and interoperability in the frame of Industry 4.0 is an area usually underestimated.

Our future work will move toward four main directions. First, we will apply the proposed architectural framework for the development of software platforms for additional manufacturing operations. In particular, we will prioritize its application to quality processes in the frame of the predictive quality strategy. Second, we will focus on the user interaction aiming at achieving an optimized human-machine collaboration through explainable AI and digital intelligent assistants. Third, we will provide a taxonomy of appropriate technologies for each part of the architecture in order to facilitate the software implementation. Fourth, we will design a holistic business process management view of all the main manufacturing operations in order to further assure the organizational interoperability for the transition to Industry 4.0.

## Data Availability Statement

The raw data supporting the conclusions of this article will be made available by the authors, without undue reservation.

## Author Contributions

The review of Enterprise Integration and Interoperability, Big Data Processing, and Industry 4.0 as well as the first version of the proposed architectural framework and its instantiation to predictive maintenance was performed by AB. GM supervised and guided this research work, refined the proposed architectural framework, and made improvements to the paper. All authors contributed to the article and approved the submitted version.

## Conflict of Interest

The authors declare that the research was conducted in the absence of any commercial or financial relationships that could be construed as a potential conflict of interest.
